# Editorial: Next-Generation Cancer Therapies Based on a (R)evolution of the Biomarker Landscape

**DOI:** 10.3389/fphar.2022.861424

**Published:** 2022-03-07

**Authors:** Claudia Cerella, Anne Lorant, Katia Aquilano, Marc Diederich

**Affiliations:** ^1^ Laboratoire de Biologie Moléculaire du Cancer, Hôpital Kirchberg, Luxembourg, Luxembourg; ^2^ Laboratory of Biochemistry, Department of Biology, University of Rome Tor Vergata, Rome, Italy; ^3^ Department of Pharmacy, College of Pharmacy, Seoul National University, Seoul, South Korea

**Keywords:** cancer biomarkers, precision oncology, targeted agents, immunotherapy, therapy response prediction

Targeted and immunomodulatory agents have driven the field of cancer therapy toward precision oncology. Therapeutic protocols can now be tailored to each patient after identifying molecular alterations and vulnerabilities to provide the most case-effective therapeutic option. Even though personalized therapies have offered clinical benefits to responsive patients, they also reveal limitations (Gambardella et al., 2020; Malone et al., 2020). The multi-arm precision clinical trial NCI-MATCH (National Cancer Institute-Molecular Analysis for Therapy Choice) applied DNA sequencing to assign the most appropriate targeted therapies to individual cancer patients. As a result, 18% of the 38% of patients with an actionable mutation could benefit from such a personalized treatment; moreover, a significant proportion of them did not respond to these therapies (Flaherty et al., 2020; Commentary, 2021). Similarly, immunotherapies hold potential in cancer therapy; however, the benefit of these approaches is counterbalanced by early disease progression and frequent adverse events (AEs) in real-world experience (McKean et al., 2020). The robustness of biomarkers predicting patient response or AEs must be improved. To reach this goal, several ongoing clinical trials have been launched to validate innovative precision immuno-oncology markers with the intent to improve patient stratification and drug response prediction (NCT03833440; NCT03493581; NCT04589845; NCT03917537). Robust indicators are required to: 1) monitor and predict the cellular fates of intratumor subclones presenting heterogeneous genetic profiles and therapeutic vulnerabilities; 2) identify stem cells; 3) track cell communication within the tumor microenvironment (TME); 4) characterize determinants of metabolic plasticity and 5) cancer immune evasion. Furthermore, therapies need to be adapted. This approach requires the integration of multi-parametric models, including *in vitro*/*ex vivo* drug screening platforms, *in vivo* patient-derived models, computational methods, and retrospective/prospective cancer patient studies (Letai et al., 2021).

This special issue discusses the evolving concept of biomarkers in cancer therapy, considering the rapid evolution of the treatment landscape. The volume includes 14 contributions encompassing reviews, metadata studies, and original articles. Globally, they provide a comprehensive overview of the current classification of biomarkers, suggest innovative approaches, or rediscuss/implement the validity of biomarker-driven treatments. Discussions involve conventional and personalized therapies.

## The Evolving Concept of the Cancer Biomarkers

Diversified and innovative investigational technologies in pharmacological and medical sciences require a continuous update of biomarker classification. Worldwide medical agencies are developing guidelines for biomarker qualifications (e.g., the FDA-NHI Biomarker Working Group, https://www.fda.gov/about-fda/center-drug-evaluation-and-research-cder/fda-biomarkers-working-group; the EMA Concept Paper EMA/CHMP/800914/2016, https://www.ema.europa.eu/en/predictive-biomarker-based-assay-development-context-drug-development-lifecycle). The field of cancer biomarkers mirrors this dynamic scenario. Louie et al. provide a comprehensive overview of the evolving field of cancer biomarkers. After defining the different categories, the authors discuss their clinical application and utility by examples. The article integrates the contribution of different technologies to facilitate the discovery of cancer biomarkers, ranging from omics assays (genomics, transcriptomics, proteomics, and metabolomics) to the most recent approaches (machine learning, analysis of tissues, biological fluids, and liquid biopsies).

## Biomarkers for Personalized Immuno-Oncology

Cancer immunotherapy drives recent therapy breakthroughs. The cellular and molecular complexity of the immune system mirrors multiple subverted processes that innovative compounds can efficiently target to harness the immune response (Waldman et al., 2020). Despite this exciting premise, most patients do not respond to immunotherapies while developing severe AEs. Although some alterations are associated with immunotherapy response, the underwhelming therapeutic outcomes indicate the limited predictive power of most of these putative response biomarkers (McKean et al., 2020). Tian et al. describe the lack of prediction of T cell exhaustion as a significant limitation of the currently used indicators of response to immune checkpoints inhibitors (ICIs), like the microsatellite instability/stability (MSI/MSS) status or the tumor mutational burden (TMB). Consequently, they developed the TMEPRE computational method, which integrates two scores respectively measuring the level of T cell infiltration in the TME (TME1. TCellInfiltration) and their ability to respond to ICIs (TME2. CellResponse). Their approach, specific for colorectal cancer (CRC), matches the expected percentages of responders among MSI or MSS CRC, providing mechanistic insights about their resistance. Abdolahi et al. investigate the antitumor potential of *ex vivo*-expanded, IL-2 activated NK cells combined with an anti-PD1 antibody (Nivolumab) using a xenograft model of gastric cancer. The authors show that anti-PD1 treatment improves the efficacy of adaptive NK cell therapy by using an integrated analysis including morphometric, immunohistochemical, and flow cytometric analyses. A maximal response was achieved when anti-PD1-pretreated NK cells were injected. Interested readers will find a comprehensive and up-to-date overview of clinically approved and investigational ICIs in the review article of Lee et al. Each ICI description comprises the molecular structure, the mechanism of action, cell expression pattern, targeting agents, and ongoing clinical trials, further summarized in accompanying tables.

## Maximizing the Clinical Benefits in Cancer Therapy

Improving responder prediction and progressively adapting therapies remain urgent needs. Nikanen et al. use an *ex vivo* drug screening platform as a functional diagnostic method for therapy decision-making. They report a case study of a patient affected by a metastatic parotid squamous cell carcinoma, a rare and aggressive type of cancer generally diagnosed at an advanced stage. They combined a phenotypic-based assay with a reverse-phase protein array (RPPA) drug screening using 318 anti-cancer agents. They applied this setup on tumor cells isolated in two stages to adapt the treatment to the disease progression. They further improved the control of the disease by the off-label use of drugs providing the most efficient *ex vivo* results. AEs cause therapy discontinuation. Tawk et al. reflect on current strategies to minimize morbidities by optimizing treatment intensity. Human papillomavirus (HPV)-driven head and neck squamous cell carcinoma (HNSCC) is the topic of this overview. The authors suggest that a deeper molecular characterization of the HNSCC TME may identify new biomarkers to be validated in next-generation de-escalation trials.

## Implementing the Prognostic/Predictive Potential of Cancer Biomarker

Protocol conditions are critical when establishing the potential of biomarkers. Ungureanu et al. performed a meta-analysis of the clinicopathological relevance of claudin (CLDN) 18.2 expression in gastric cancer. The authors did not establish significant correlations between CLDN 18.2 and clinical features (including TNM stages, Laurent classification, human epidermal growth factor receptor 2 (HER), grading, and overall survival (OS)) when using two different cutoff values to classify CLDN 18.2 positivity. However, higher CLDN 18.2 expression could be observed in specific T/N stages when the cutoff for CLDN 18.2 positivity was set higher. The authors predict that a re-evaluation of classification criteria (e.g., more specific assays for staining and quantification and the cutoff threshold for CLDN 18.2 positivity) might improve the CLDN 18.2 prognostic value. Hsiao et al. aim at validating c-Myc expression levels as a new marker of resistance to the “7 + 3” induction regimen of *de novo* acute myeloid leukemia (AML) patients. They used the complete remission (CR) rates of a cohort of 75 patients from one prospective and one retrospective study as a readout. They discovered that patients unable to reach a CR display higher c-Myc gene expression levels. Of note, responder prediction is facilitated by combining c-Myc positivity to high-risk cytogenetics. This study establishes the gene (but not the protein) expression level combined with the cut-off of expression positivity as critical determinants for consistent results.

## Clinicopathological Significance of Specific Alteration Patterns

Tian et al. review the dual role of the integrated stress response (ISR) on cell survival/death and autophagy. The authors discuss strategies to manipulate the ISR to sensitize tumor cells to specific agents (protease and tyrosine kinase inhibitors, ISR activators, and ICIs). Raufi et al. discuss the role of autophagy in pancreatic ductal carcinoma (PDAC). In this aggressive type of cancer, autophagy is upregulated and contributes to carcinogenesis and therapy resistance. Functional studies document the dependency of PDAC on this process to sustain metabolism and modulate immunity. Mechanistically, a hypoxic TME promotes autophagy and the unfolded protein response (UPR) as adaptive responses to ISR. The authors suggest MEK inhibitors and ICIs targeting the ISR as promising candidates for combinatorial therapies. This article provides extensive tables summarizing autophagy inhibitors and overviewing clinical trials with autophagy modulators in PDAC. The lack of biomarker-driven treatments in selected cancer types is a major challenge. Liguori et al. discuss the therapeutic potential of the ISR as a pharmacological target. The authors investigate the effect of preclinical drug candidates against small cell lung cancer (SCLC) without actionable biomarkers. Chang et al. review chromosomal rearrangements in pediatric solid tumors outside the central nervous system (CNS). Promising therapeutic regimens and ongoing clinical trials are reported for each type of cancer.

The aberrant regulation of lipid metabolism causes carcinogenesis and therapy resistance (Bacci et al., 2021). Tomacha et al. characterize the metabolic profile of 155 cholangiocarcinoma (CCA) patients, identifying an inverse correlation between fatty acid synthase (FASN) expression and OS. FASN knockdown inhibits CCA cell proliferation and survival, while metabolomics suggests the purine metabolism as the most relevant pathway affected by FASN knockdown. Approaches targeting FASN might thus represent a potential strategy for this aggressive type of cancer.

## Beyond Gene and Protein Biomarkers

Gene or protein expression networks commonly constitute prognostic signatures. Li et al. analyze the potential of long coding RNAs (lncRNAs) in the prognosis of papillary thyroid cancer (PTC). Using four different databases, the authors identify 5 promising hub lncRNAs and develop two prognostic risk models for PTC OS and disease-free survival (DFS) based on lncRNA-miRNA-mRNA competing endogenous RNA (ceRNA) network. The resulting connectivity Map predicts candidate compounds for PTC treatment.

Overall, this special issue provides new ideas of cancer biomarkers and offers a discussion forum to design and improve clinical trials and validate novel biomarkers predictive of therapy response and optimization. We thank all authors for their valuable contributions.
